# Genito-Urinary Therapeutics

**Published:** 1881-12

**Authors:** 


### GENITO-URINARY THERAPEUTICS.

We hope to commence, next month, a series of articles on this subject. The notes on each remedy will be arranged on a plan somewhat similar to that of Bell's work on Diarrhœa. None but reliable symptoms will be given; having the assistance of some of our best therapeutists in compiling these notes, they cannot fail to be valuable.

